# Invalid Passengers on Board Ship

**Published:** 1886-10-30

**Authors:** 


### THE EDITOR'S LETTER-BOX.

[Our Correspondents are reminded that prolixity is a great bar to publication, and that brevity of style and conciseness of statement greatly facilitate early publication.]

### INVALID PASSENGERS ON BOARD SHIP.

A SISTER writes from Edinburgh: "It has occurred to me that some of your readers might be able to give me some information with regard to a report which I have heard of trained nurses being engaged to attend upon the invalid passengers on board vessels. I am anxious to go on a voyage, and should be glad to hear particulars, as I do not know anyone to ask about it. I know that there are often invalid ladies going abroad who require a nurse, but it is difficult to hear of them just when you wish. Can anyone tell me what would be the duties of a nurse on board ship, her possible salary, and where I ought to apply to hear of such situations?"

*** Can any of our readers answer Sister's questions?—ED. T. H.

